# The job competency of radiological technologists in Korea based on specialists opinion and questionnaire survey

**DOI:** 10.3352/jeehp.2017.14.9

**Published:** 2017-05-11

**Authors:** Chang-Seon Lim, Yang-Sub Lee, Yong-Dae Lee, Hyun-Soo Kim, Gye-Hwan Jin, Seong-Youl Choi, Yera Hur

**Affiliations:** 1 Department of Radiological Science, Konyang University College of Medical Sciences, Daejeon, Korea; 2 Department of Radiology, Asan Medical Center, Seoul, Korea; 3 Department of Diagnostic Radiology, Inje University Sanggye Paik Hospital, Seoul, Korea; 4 Department of Radiological Technology, Shingu College, Korea; 5 Department of Radiology, Nambu University, Korea; 6 Department of Occupational Therapy, Kwangju Women’s University, Gwangju, Korea; 7 Department of Medical Education, Konyang University College of Medicine, Daejeon, Korea; Hallym University, Korea

**Keywords:** Licensure, Professionalism, Republic of Korea, Safety, Radiological technology

## Abstract

**Purpose:**

Although there are over 40,000 licensed radiological technologists (RTs) in Korea, job competency standards have yet to be defined. This study aims to clarify the job competency of Korean RTs.

**Methods:**

A task force team of 11 professional RTs were recruited in order to analyze the job competency of domestic and international RTs. A draft for the job competency of Korean RTs was prepared. A survey was then conducted sampling RTs and the attitudes of their competencies were recorded from May 21 to July 30, 2016.

**Results:**

We identified five modules of professionalism, patient management, health and safety, operation of equipment, and procedure management and 131 detailed job competencies for RTs in Korea. “Health and safety” had the highest average score and “professionalism” had the lowest average score for both job performance and importance. The content validity ratios for the 131 subcompetencies were mostly valid.

**Conclusion:**

Establishment of standard guidelines for RT job competency for multidisciplinary healthcare at medical institutions may be possible based on our results, which will help educators of RT training institutions to clarify their training and education.

## Introduction

The professional standards of competency for radiological technologists (RTs) should be identified for upholding professional conduct within the field. The International Society of Radiographers and Radiological Technologists (ISRRT), in addition to countries such as the United States, have previously outlined the professional standards for RTs [1,2,3,4]. Nonetheless, the job competencies for Korean RTs have not been identified yet. In Korea, since 1961, 41,404 RTs have been issued a practicing license [5]. However, only a few studies have been performed on the job analysis [6]. To the best of our knowledge, no studies were found on the subject of RTs job analysis from the search engine of Korean National Assembly Library [7]. Using the key word “radiological technologist job competency” in the PubMed Central, ten articles were found [8], but no articles dealt with the RTs job analysis.

Job competency comprises the standards of education, components of the licensing examination, and contents of the certification training program [9,10,11]. Therefore, our study aimed to clarify the job competency of Korean RTs and focus on the following three overarching questions: first, what are the core competencies required for Korean RTs?; second, what are the sub-competencies required for Korean RTs?; and third, are the identified competencies for RTs valid?

## Methods

### Study design

This was a cross sectional study including a descriptive analysis. For the purpose of this study, we recruited a task force team of professional RTs with an average of over 20 years of radiology-related educational or practical experience, which included the following 11 members: the president, four vice presidents, and two directors of the KART (Korean Radiological Technologist Association); three professors in radiological technology; and a manager of RTs in a general hospital. We consulted the team 7 times to evaluate the current guidelines outlined for the competencies of RTs as indicated in the ISRRT of Australia, United States and Canada, and evaluated the Korean RTs job analysis [6]. We studied with the team the “guidelines for the education of entry-level professional practice in medical radiation sciences” of the ISRRT, the “competency requirements” of the ARRT (American Registry of Radiologist) [2]. We closely examined the five standards, 20 descriptors, and 69 outcomes [3] of the AIR (Australian Institute of Radiography). The CAMRT (Canadian Association of Medical Radiation Technologists) had 5 modules, 20 sections, and 131 competencies [4], but we were not able to find any validity results of these competencies.

The team categorized the competencies of Korean RTs into the following: core competency, sub-competency, and more detailed competency. Subsequently, a survey (Appendix 1) was developed and distributed, from May 21 to July 30, 2016, to RT professors, RT managers in large healthcare organizations, and general RTs to evaluate the validity of the recommended competencies outlined by the team.

### Materials and subjects

Survey items related to the job competency of domestic and international RTs were analyzed. The results were subsequently used to prepare a draft for the job competency of Korean RTs. The survey was then disseminated to RTs, with the expertise and attitudes of job competency specialists additionally acquired.

The core competencies required for Korean RTs are categorized into five elements: “professionalism,” “patient management,” “health and safety,” “operation of equipment,” and “procedure management.” The five core competencies were further subdivided into 24 sub-areas, and then further into 131 specific competencies.

The survey questions were assessed using a 5-point Likert scale: 1= not at all valid, 2= not valid, 3= neutral, 4= valid, and 5= very much valid. The surveys were distributed to each hundred RT professors, RT managers in large healthcare organizations and general health institutions. Overall, 175 out of 300 questionnaires were returned: 45, 43, and 87 questionnaires were returned from each of the above survey groups, respectively. After excluding inappropriate responses such as missing data, 147 answers were used for the final statistical analysis.

### Statistics

The survey results evaluating the job competency of Korean RTs were analyzed using PASW SPSS Statistics ver. 18.0 (SPSS Inc., Chicago, IL, USA). Differences in the mean score between the importance and work performance for the core competency and sub-competency were analyzed by Independent-sample t-test. Korean RTs were first analyzed for the distribution frequency, and the mean results were recorded. The content validity ratio (CVR) and Cronbach’s α of each job competency were calculated by analyzing the mean, standard deviation, quartile, and median. The Cronbach’s α of the survey results was 0.99. The CVR was calculated by the following formula.

$$CVR=\frac{n_{e}-\frac{N}{2}}{\frac{N}{2}}$$

$${\text{N}}_{\text{e}}\text{:number of the valid (point 4,5) }$$

### Ethical approval

Students’ informed consents were obtained.

## Results

### Characteristics of the respondents of the questionnaire survey

The survey respondents included Korean RT professors, RT team managers, and RTs working as general technicians. The mean number of years for either working or teaching within the profession was over 10 years, as shown in Table 1. RTs from primary or secondary hospitals constituted 49.5% of the respondents, and 50.5% of the respondents were working at tertiary hospitals. Raw data were available from Supplement 1.

### Collective opinions of the survey respondents

*The performance and rated importance of the core competencies of Korean RTs:* The analysis of the core competency of Korean RTs and the level of importance for which they were rated showed an overall mean score of 4.412 points for importance and 4.133 points for performance (P< 0.001). A statistically higher score was attributed to the importance of the five core competencies compared to the work performance of the respondents for the same criteria. “Health and safety” was indicated as being the most important, followed by “procedure management,” “operation of equipment,” “patient management,” and “professionalism” (Table 2). “Health and safety” had the highest score for the performance criteria.

*The performance and importance for the sub-competencies of Korean RTs:* The scores for the rated importance and performance of each of the sub-competencies of Korean RTs were comparatively analyzed. The results of the comparison for the sub-competencies and the level of importance assigned by the Korean RTs are shown in Table 3. With the exception of sub-competency, “A.6: use of resources,” the remainder of the 24 sub-competencies showed significantly higher scores for importance than for performance. The most important sub-competency was “C.3: radiation safety practices” (mean= 4.729, P= 0.000), whereas “B.2: patient safety” (mean= 4.404, P= 0.015) had the highest score for performance.

*CVR of detailed competencies:* A CVR of 0.33 was defined as the minimum for which a score was considered valid; a ratio lower than 0.33 indicated that the competency was not valid. CVR analysis was performed with the subdivided competencies for an increased accuracy. The invalidity ratio from each panel category was considered, but the overall total ratio was factored into the final decision. Therefore, the result indicated that all five core modules consisted of valid competencies, and 131 sub-competencies showed positive results except for two sub-competencies scored as invalid competencies by the statistical analysis, which were “B.3.7: perform venipuncture” (CVR= 0.197) and “E.3.5: perform rectal tube insertion” (CVR= 0.306). Sub-competencies such as “applying ALARA (as low as reasonably achievable) principle” (CVR= 0.986), “apply knowledge of radiation effects and risks” (CVR= 0.959), and “use protective devices and apparel for personnel” (CVR= 0.959) showed high valid CVR results (Table 4).

## Discussion

All the CVR scores turned out to be appropriate in all five modules; module A (professionalism) showed 0.719 mean CVR score, module B (patient management) 0.754, module C (health and safety) 0.845, module D (operation of equipment) 0.766, and module E (procedure management) 0.767. Also, most of the categories showed appropriate CVR values, ranging from the highest score of 0.986 (C.3.1) to the lowest score of 0.415 (B.3.11), except for two categories. B.3.7 (perform venipuncture necessary for the test) and E.3.5 (perform rectal tube insertion) were indicated to be inappropriate responsibilities for RTs. However, with regard to the category B.3.7 (perform venipuncture necessary for the test), the surveyed professors indicated that venipuncture should be warranted a CVR value of 0.526, while the RT managers and general RTs indicated that such measures would be inappropriate with CVR values of 0.103 (RT managers) and 0.075 (general RTs). This implied a difference in opinion between academics and clinical RTs. With regards to the category E.3.5 (perform rectal tube insertion), the surveyed professors indicated that rectal tube insertion would be appropriate with a CVR value of 0.526, while the RT managers and general RTs responded that the procedure would be inappropriate with CVR values of 0.310 (RT managers) and 0.200 (general RTs), thereby showing a difference in opinion between academics and clinical RTs.

In addition, there were slight differences in opinions of each of the professional groups with regards to the overall appropriateness of some of the detailed competencies. The general RT group indicated that two of the detailed competencies B.3.7 (perform venipuncture necessary for the test) and E.3.5 (perform rectal tube insertion) were clinically inappropriate. These competencies were categorized as invasive practices at the time of the evaluation during the certification of medical institutions in Korea [13]. Therefore, these procedures are delegated to either physicians or nurses in Korea, despite being listed as a responsibility of RTs internationally, such as in Canada [4,14]. Accordingly, there appears to be a difference in opinion between the clinical RTs and the academics.

Although the responsibilities of RTs are usually limited to radiation tests, treatment for these procedures have been restricted to fundamental concepts, requiring further evaluation through national licensing examinations [15,16,17]. Therefore, this study has highlighted and provided further insight into the previously poorly characterized aspects of the field, such as the attitudes of specialist RTs and other aspects including legal and ethical qualifications, interdisciplinary communication, infection control, drug control, and others.

Further research, in addition to more detailed definition and establishment of the work competencies of Korean RTs, is necessary as this would form the foundation for the education of prospective students and for the improvement of the license examination process. Additionally, this would also improve the work place efficacy for Korean RTs at medical institutions.

There are some limitations in this study and further considerations to be noted. In Korea, studies on the job competency of RTs are lacking. The response rate of the surveys in our study was only 58%, which may be due to the fact that the surveys contained too many questions for the respondents to answer promptly. We were also unable to establish the sub-competencies for each specialized field, since many studies only focused on the more common competencies of RTs who work in radiology, nuclear medicine, and radiotherapy. Future studies on the sub-competencies for each specialized field should be performed on the basis of our study results.

In conclusion, we attempted to establish a standard guideline for RTs in Korea. Having provided the basic outline of RT job competency, our results will help the educators of RT training institutions to clarify their training and educational content which will lead to more appropriately skilled RTs.

## Figures and Tables

**Table 1.** General characteristics of the survey respondents, categorized as RT professors, managers, and general RTs

Variable	RT professors (n = 38)	RT managers (n = 29)	General RTs (n = 80)	Total (N = 147)
Gender				
Men	36 (94.7)	28 (96.6)	60 (75.0)	124 (84.4)
Women	2 (5.3)	1 (3.4)	20 (25.0)	23 (15.6)
Age (yr)	48.84 ±7.08	51.86±5.32	40.34±7.82	44.76±8.74
Degree				
Bachelor (college)	0	8 (27.6)	22 (27.5)	30 (20.4)
Bachelor (university)	1 (2.6)	6 (20.7)	40 (50.0)	47 (31.0)
Masters	5 (13.2)	13 (44.8)	15 (18.8)	33 (22.4)
Doctoral	32 (84.2)	2 (6.9)	3 (3.8)	37 (25.2)
Teaching area				
Theory	10 (26.3)	-	-	10 (26.3)
Practice	7 (18.4)	-	-	7 (18.4)
Theory & practice	21 (55.3)	-	-	21 (55.3)
Years of teaching experience	11.74±9.58	-	-	11.74±9.58
Affiliation				
Tertiary hospital	-	16 (55.2)	39 (48.8)	55 (50.5)
Secondary hospital	-	11 (37.9)	39 (48.8)	50 (45.9)
Primary hospital	-	1 (3.4)	1 (1.3)	2 (1.8)
Health center	-	1 (3.4)	1 (1.3)	2 (1.8)
Years of working experience	13.26±6.90	27.11±5.22	15.59±7.78	17.33±8.67

Values are presented as number (%) or mean±standard deviation. RT, radiological technologist.

**Table 2.** The mean and standard deviation for the criteria of importance and work performance for the core competencies

Module	Core competency	Importance	Performance	t-value^a)^	P-value
Module A	Professionalism	4.302±0.525	4.064±0.610	3.566	<0.001
Module B	Patient management	4.393±0.504	4.201±0.588	2.981	0.003
Module C	Health and safety	4.523±0.463	4.210±0.690	4.516	< 0.001
Module D	Operation of equipment	4.396±0.581	4.070±0.738	4.16	< 0.001
Module E	Procedure management	4.465±0.510	4.106±0.671	5.089	< 0.001
Total mean		4.415±0.468	4.133±0.612	4.406	< 0.001

Values are presented as mean±standard deviation. RT, radiological technologist.

a) t statistics among RT professors, RT managers, and general RTs.

**Table 3.** The level of importance and work performance for the sub-competencies of Korean radiological technologists

No.	Sub-competency	Importance	Performance	t-value^a)^	P-value
A.1	Legal and ethical requirements	4.570±0.518	4.357±0.661	3.054	0.002
A.2	Professional behavior	4.375±0.502	4.077±0.657	4.313	0.000
A.3	Communication	4.207±0.632	3.967±0.626	3.241	0.001
A.4	Decision making	4.329±0.676	4.080±0.764	2.938	0.004
A.5	Interprofessional practice	4.361 ±0.621	4.133±0.724	2.876	0.004
A公	Use of resources	4.154±0.730	4.033±0.757	1.387	0.166
A.7	Quality assurance	4.401±0.572	4.131±0.766	3.386	0.001
A.8	Research	4.023±0.743	3.704±0.871	3.339	0.001
B.1	Patient interactions	4.400±0.535	4.182±0.615	3.221	0.001
B.2	Patient safety	4.562±0.487	4.404±0.600	2.457	0.015
B.3	Patient assessment and care	4.218±0.683	4.019±0.735	2.387	0.018
C.1	Infection control and materials	4.375±0.645	4.089±0.817	3.291	0.001
C.2	Self-protection	4.463±0.604	4.168±0.786	3.557	0.000
C.3	Radiation safety practices	4.729±0.402	4.341±0.768	5.346	0.000
C.4	Radiation safety education	4.478±0.572	4.240±0.713	3.110	0.002
C.5	Emergency procedures	4.571±0.597	4.206±0.883	4.103	0.000
D.1	Principles of radiological technology equipment	4.238±0.719	3.924±0.842	3.411	0.001
D.2	Image acquisition and management	4.377±0.648	4.166±0.747	2.566	0.011
D.3	Equipment quality control	4.429±0.690	4.067±0.892	3.847	0.000
D.4	Image quality	4.478±0.572	4.240±0.713	3.110	0.002
D.5	Other imaging modalities	4.457±0.683	3.956±1.030	4.850	0.000
E.1	Clinical principles	4.578±0.520	4.186±0.698	5.380	0.000
E.2	Imaging procedures	4.488±0.590	4.200±0.683	3.806	0.000
E.3	Pharmaceutical administration	4.329±0.621	3.931±0.821	4.614	0.000

Values are presented as mean±standard deviation. RT, radiological technologist.

a) t statistics among RT professors, RT managers, and general RTs.

**Table 4.** The CVR for the competency elements

No.	Subdivision of competency elements	Level of importance						
		Total				Professors	Managers	RTs
		Mean ± standard deviation	Median	CVR	Cronbach’s α	CVR	CVR	CVR
A.2.9	Utilize basic conflict management techniques	4.12±0.85	4.0	0.483	0.988	0.211	0.517	0.600
A.2.11	Provide constructive feedback to others	4.13±0.86	4.0	0.524	0.988	0.263	0.655	0.600
A.8.3	Participate in activities that require application of research methodology	3.97±0.82	4.0	0.456	0.988	0.316	0.517	0.500
B.3.7	Perform venipuncture	3.50±1.50	4.0	0.197	0.988	0.526	0.103	0.075
B.3.8	Assist with administration of pharmaceuticals	4.02±1.10	4.0	0.429	0.988	0.263	0.379	0.519
B.3.11	Provide patient interventions (assist with administration of oxygen, suction, monitor) vital signs etc.	3.97±1.24	4.0	0.415	0.988	0.316	0.172	0.550
E.3.5	Perform rectal tube insertion	3.72±1.38	4.0	0.306	0.988	0.526	0.310	0.200

CVR, content validity ratio; RT, radiological technologist.

## Supplementary materials

Supplement 1. Data file is available from https://dataverse.harvard.edu/dataset.xhtml?persistentId=doi:10.7910/DVN/GGKTMC

Supplement 2. Audio recording of the abstract.

## Appendix 1. Job competencies for radiological technologists in Korea

**Table:**

Subordinate	No.	Elements
Module A: professionalism		
A.1: legal and ethical requirements	A.1.1	Practice within Medical Technologists, Etc. Act.
	A.1.2	Comply with national legislation and regulations affecting the practice of medical radiation technology.
	A.1.3	Comply with requirements of provincial regulatory body, including applicable standards of practice and sexual abuse prevention guidelines.
	A.1.4	Practice within the Korean Radiological Technologist Association or national association code of ethics, as relevant.
	A.1.5	Practice in a manner that recognizes the patient’s legal rights.
A.2: professional behavior	A.2.1	Present a professional appearance and manner.
	A.2.2	Interact respectfully with others.
	A.2.3	Provide care in an unbiased manner.
	A.2.4	Practice within limits of personal knowledge and skills.
	A.2.5	Comply with organizational policies and directives.
	A.2.6	Maintain thorough and complete workplace documentation.
	A.2.7	Respond professionally to changes impacting the practice environment.
	A.2.8	Utilize techniques to manage personal stress in the workplace.
	A.2.9	Utilize basic confl t management techniques.
	A.2.10	Respond professionally to feedback received from others.
	A.2.11	Provide constructive feedback to others.
	A.2.12	Provide information and guidance to students in the medical radiation technology workplace.
	A.2.13	Engage in refl tive practice.
	A.2.14	Implement a learning plan to enhance personal knowledge and skills.
	A.2.15	Demonstrate basic knowledge of current and emerging issues in health care relevant to the practice of medical radiation technology.
	A.2.16	Demonstrate basic knowledge of current and emerging practices and technological developments in the fi of medical radiation technology.
A.3: communication	A.3.1	Use effective written communication skills.
	A.3.2	Use effective oral communication skills.
	A.3.3	Use effective interpersonal skills.
	A.3.4	Utilize medical terminology in professional communication.
	A.3.5	Explain complex and technical matters related to medical radiation technology to the level of the respondent’s understanding.
A.4: decision	A.4.1	Appraise decision options based on best practice evidence, clinical information, resource implications, and other contextual factors.
making	A.4.2	Use professional judgment to reach decisions.
	A.4.3	Take responsibility for decisions and actions.
A.5: inter-profes- sional practice	A.5.1	Recognize the roles of health care professionals commonly encountered in the medical radiation technology workplace.
	A.5.2	Contribute productively to teamwork and collaborative processes.
	A.5.3	Contribute knowledge of medical radiation technology in collaborative practice.
A.6: use of resources	A.6.1	Prioritize workfl w to optimize patient care.
	A.6.2	Prioritize workfl w to optimize use of resources.
	A.6.3	Monitor inventory of materials and supplies, and respond accordingly.
A.7: quality assur- ance	A.7.1	Maintain awareness of factors in the clinical environment that may affect delivery of care, and take appropriate action.
	A.7.2	Participate in activities that support a quality assurance program.
	A.7.3	Apply principles of risk management.
A.8: research	A.8.1	Demonstrate basic knowledge of research methodology and ethics.
	A.8.2	Critically appraise professional literature to assess relevance to practice.
	A.8.3	Participate in activities that require application of research methodology.
Module B: patient management		
B.1: patient interac- tions	B.1.1	Respect the dignity, privacy, and autonomy of the patient.
	B.1.2	Maintain professional boundaries.
	B.1.3	Recognize and respond appropriately to cultural, religious, and socio-economic variables affecting patient management.
	B.1.4	Adapt interactions to enhance communication with patient and support persons.
	B.1.5	Provide complete information about procedures to patient and support persons, and verify understanding.
	B.1.6	Respond to questions from patient and/or support persons, or direct them to appropriate personnel.
	B.1.7	Ensure ongoing, informed consent to procedures.
B.2: patient safety	B.2.1	Ensure a safe physical environment.
	B.2.2	Verify patient identity.
	B.2.3	Verify the accuracy and completeness of pre-procedure documentation.
	B.2.4	Transport patient safely.
	B.2.5	Transfer patient safely.
	B.2.6	Utilize immobilization devices.
	B.2.7	Ensure proper function of the patient’s supportive devices and equipment.
	B.2.8	Assess and respond to any changes in patient condition.
	B.2.9	Recognize medical emergencies, and respond.
	B.2.10	Ensure post-procedure transfer of care.
	B.2.11	Verify accuracy and completeness of post-procedure documentation.
	B.2.12	Ensure entry of information to data archiving system.
B.3: patient assess- ment and care	B.3.1	Enhance patient comfort.
	B.3.2	Review clinical history provided, relative to requested procedure, and address discrepancies.
	B.3.3	Obtain information from patient or support person.
	B.3.4	Identify clinically relevant details, and respond.
	B.3.5	Determine the patient’s pregnancy status and respond.
	B.3.6	Assess patient for contraindications to procedure and respond.
	B.3.7	Perform venipuncture.
	B.3.8	Assist with administration of pharmaceuticals.
	B.3.9	Adapt procedures based on the patient’s physical and cognitive condition.
	B.3.10	Provide care for the patient's physiological needs.
	B.3.11	Provide patient interventions.
	B.3.12	Advise patient of necessary post-procedure follow-up.
Module C: health and safety		
C.1: infection con- trol and materi- als handling	C.1.1	Employ routine practices for infection control.
	C.1.2	Employ transmission-based precautions.
	C.1.3	Follow standardized procedures for patients with compromised immunity.
	C.1.4	Use aseptic technique.
	C.1.5	Use sterile technique.
	C.1.6	Follow standardized procedures for handling and disposing of sharps, and contaminated and biohazardous materials.
C.2: self-protection	C.2.1	Utilize protective equipment.
	C.2.2	Employ proper body mechanics.
	C.2.3	Ensure a safe working environment.
C.3: radiation safety practices	C.3.1	Apply ALARA (as low as reasonably achievable) principle.
	C.3.2	Apply knowledge of radiation effects and risks.
	C.3.3	Use protective devices and apparel for personnel.
	C.3.4	Implement safe practices to minimize radiation dose to personnel and support persons.
	C.3.5	Implement safe practices to minimize radiation dose to patients.
	C.3.6	Monitor personal radiation exposure, and respond.
C.4: radiation safety education	C.4.1	Provide information regarding radiation risks and safe practices.
	C.4.2	Provide education regarding organ sensitivities and safe practices.
C.5: emergency procedures	C.5.1	Recognize emergency situations involving equipment and respond accordingly.
Module D: operation of equipment		
D.1: principles of radiological technology equipment	D.1.1	Apply knowledge of radiation physics.
	D.1.2	Apply knowledge of operational components of imaging systems.
	D.1.3	Apply knowledge of radiation interactions.
	D.1.4	Apply knowledge of computer technology.
D.2: image acquisi- tion & manage- ment	D.2.1	Operate imaging systems listed.
	D.2.2	Select and optimize parameters for performing a procedure.
	D.2.3	Utilize common accessory equipment listed.
	D.2.4	Activate, monitor, and manage acquisition.
	D.2.5	Perform post-processing on acquired image data.
	D.2.6	Utilize digital networking and archiving system.
	D.2.7	Evaluate images for the purpose of reject analysis.
D.3: equipment quality control	D.3.1	Assess performance of imaging equipment.
	D.3.2	Assess performance of accessory equipment.
D.4: image quality	D.4.1	Apply knowledge of principles affecting image quality.
	D.4.2	Evaluate diagnostic quality of image, and respond.
	D.4.3	Verify accuracy of patient demographics.
	D.4.4	Verify visibility and accuracy of radiographic markers and annotations.
	D.4.5	Evaluate image for artifacts, and respond.
D.5: other imaging modalities	D.5.1	Apply knowledge of basic principles of positron emission tomography-computed tomography.
	D.5.2	Apply knowledge of basic principles of magnetic resonance imaging.
	D.5.3	Apply knowledge of basic principles of diagnostic ultrasound.
	D.5.4	Apply knowledge of basic principles of single-photon emission computed tomography-computed tomography.
Module E: procedure management		
E.1: clinical princi- ples	E.1.1	Apply knowledge of gross anatomy, relational anatomy, and physiology related to the imaging of anatomical structures.
	E.1.2	Differentiate anatomical structures on images.
	E.1.3	Apply knowledge of pathologies, anomalies, and conditions.
	E.1.4	Apply knowledge of imaging procedures and protocols in various clinical environments and modalities.
	E.1.5	Apply knowledge of the effects of pharmaceutical agents as they relate to procedures.
E.2: imaging proce- dures	E.2.1	Plan imaging procedures utilizing data available from clinical information, reports, and previous diagnostic studies.
	E.2.2	Position patient for imaging procedures, utilizing anatomical landmarks and relational anatomy.
	E.2.3	Adapt positioning in response to patient condition and clinical environment.
	E.2.4	Adapt protocol in response to patient condition and clinical environment.
	E.2.5	Align imaging system to demonstrate required anatomical structure(s).
	E.2.6	Distinguish patterns consistent with normal results and normal variants.
	E.2.7	Recognize patterns consistent with abnormal results and pathologies
	E.2.8	Recognize conditions requiring urgent action and respond.
	E.2.9	Evaluate results to determine if further images are required.
E.3: pharmaceutical administration	E.3.1	Assess patient for contraindications to contrast media, and respond.
	E.3.2	Prepare contrast media.
	E.3.3	Administer contrast media via appropriate route.
	E.3.4	Prepare and administer pharmaceutical agents.
	E.3.5	Perform rectal tube insertion.
